# Identification and Characterization of a Germline Mutation in CARD11 From a Chinese Case of B Cell Expansion With NF-κB and T Cell Anergy

**DOI:** 10.3389/fimmu.2021.676386

**Published:** 2021-09-07

**Authors:** Peiwei Zhao, Qingjie Meng, Yufeng Huang, Lei Zhang, Sukun Luo, Xiankai Zhang, Li Tan, Aifen Zhou, Hao Xiong, Xuelian He

**Affiliations:** ^1^Precision Medical Center, Wuhan Children’s Hospital (Wuhan Maternal and Child Healthcare Hospital), Tongji Medical College, Huazhong University of Science & Technology, Wuhan, China; ^2^Department of Clinical Laboratory, Wuhan Children’s Hospital (Wuhan Maternal and Child Healthcare Hospital), Tongji Medical College, Huazhong University of Science & Technology, Wuhan, China; ^3^Department of Hematology & Oncology, Wuhan Children’s Hospital (Wuhan Maternal and Child Healthcare Hospital), Tongji Medical College, Huazhong University of Science & Technology, Wuhan, China

**Keywords:** BENTA, gain-of-function, lymphocytosis, NF-κB, CARD11

## Abstract

B cell expansion with NF-κB and T cell anergy (BENTA) is a rare primary immunodeficiency disorder caused by gain-of-function (GOF) mutations in the *CARD11* gene. Affected patients present with persistent B cell lymphocytosis in early childhood paired with lymphadenopathy and splenomegaly. Until now only six activating mutations from 14 patients have been reported in *CARD11*. Here we report a patient from China with polyclonal B cell lymphocytosis and frequent infections in early life. A heterozygous mutation (c.377G>A, G126D) in exon 5 of *CARD11* gene (NM_032415) was identified by whole exome sequencing. *In vitro* functional studies showed that the G126D mutation is associated with increased expression of CARD11 and NF-κB activation in Hela cells. Flow cytometry analysis indicated NK cell activity and CD107a degranulation of the patient were decreased. RNA sequencing analysis showed that a number of genes in NF-κB pathway increased while those involved in NK cell activity and degranulation were down-regulated. In summary, our work identified a *de novo* germline GOF mutation in *CARD11* with functional evidence of BENTA.

## Introduction

CARD11 (Caspase recruitment domain 11), a scaffolding protein, is highly expressed in hematopoietic tissue and lymphocytes and required for B-cell receptor (BCR) and T-cell receptor (TCR) signaling to activate Nuclear factor (NF)-κB, c-Jun N-terminal kinase (JNK), and mammalian target of rapamycin (mTOR) pathways (1, 2). Particularly, NF-κB represents a family of transcription factors that governs cell survival, proliferation, and immune response in many cell types (3, 4), wherein overactive NF-κB is often associated with B cell malignancy (5).

CARD11 is comprised of various defined domains, including N-terminal CARD (1-110), LATCH (112-130), coiled-coil (CC, 130-449), auto-inhibitory (ID, 450-666) domains, and a C-terminal MAGUK domain (667-1140), the last one of which has 3 subdomains: PSD95/ZO-1, SH3, and guanylate kinase (GUK) domains (6). Somatic CARD11 mutations were oncogenic and more than 2000 mutations had been reported in various cancers, especially in diffuse larger B cell lymphoma (https://cancer.sanger.ac.uk/cosmic/search?q=CARD11). In addition, germline CARD11 mutations have been implicated in several primary immune disorders, including severe combined immunodeficiency (SCID) (OMIM 615206) caused by homozygous loss-of-function (LOF) mutations, B cell expansion with NF-κB and T cell Anergy (BENTA) (OMIM 616452) caused by heterozygous gain-of-function (GOF) mutations (7–9), and severe atopic disease (OMIM 617638) caused by heterozygous dominant negative (DN) mutations. Up to date, 7 germline homozygous LOF mutations, 15 germline heterozygous DN mutations, and 6 germline GOF mutations (C49Y, G123S, G123D, E134G, K215del, and H234Ldel235-8) have been reported (8–13). All 6 GOF mutations identified are located in the N-terminal CARD, LATCH, and CC domains.

BENTA, first reported in 2012, is a congenital lymphoproliferative and immunodeficiency disorder and patients usually present with persistent B cell lymphocytosis paired with lymphadenopathy and splenomegaly in early childhood (8). Impaired T cell proliferation is observed in these patients, increasing their susceptibility to recurrent sinopulmonary infections (14).

Herein, we report a Chinese patient with recurrent infection and B cell lymphoproliferative disorder, from whom a germline heterozygous mutation (G126D) in the *CARD11* gene was identified using whole exome sequencing (WES). We demonstrated that this mutation leads to increased NF-κB activation *in vitro* and decreased NK cell activity *in vitro*. To further investigate the underlying mechanisms, we performed RNA-seq to identify the differential gene expression patterns resulting from the disease causing mutation.

## Material and Methods

### Study Subject

This study has been approved by the institutional review board of Wuhan Children’s Hospital, Tongji Medical College, Huazhong University of Science & Technology. A patient presented with persistent upper respiratory illness and fever was recruited in this study. Upon obtaining informed consent, peripheral venous blood was withdrawn from the patient and his parents. Genomic DNA was extracted from leukocytes of whole blood samples using the QIAamp Blood DNA mini kit (Qiagen, Hilden, Germany) according to the manufacturer’s instructions. RNA was extracted using Trizol reagent (Invitrogen).

### Whole Exome Sequencing

WES and subsequent data analysis were conducted with the help of the third party medical testing laboratory (Chigene Lab, Beijing China). Candidate variants were confirmed by Sanger sequencing using self-designed primers in the patient and his parents. A conservative analysis of mutation sites was conducted using MEGA software (https://www.megasoftware.net/).

### CARD11 Gene Plasmid Construction and Cell Transfection

The wildtype *CARD11* plasmid (WT-*CARD11)* was a gift from Xin Lin (http://n2t.net/addgene:44431). To generate G126D-*CARD11* and C49Y-*CARD11* (included as a positive control), site-directed mutagenesis was performed with the oligonucleotides 5’- GCC CTA CCT GCG TCA GTA TAA GGT CAT TGA TG -3’ and 5’- GAA GGC CAC GAG GAC CTC ACG CAC TTC -3’ using overlap PCR. All positive clones were verified for the correct sequence by Sanger sequencing. Hela cells were grown in DMEM supplemented with 10% fetal bovine serum (Gibco, Thermo Fisher Scientific), and cells cultured in 6-well plate were transfected with 2 μg plasmids (pcDNA3.1, WT, WT+G126D, G126D and C49Y) using Lipofectamine 3000 (Invitrogen), respectively, according to the manufacturer’s instructions.

### Western Blot

Cells were lysed in 1% NP-40 lysis buffer (50 mM Tris, pH 7.4, 150 mM NaCl, 1 mM EDTA, 1% NP-40, and 0.5% sodium deoxycholate) for 30 min on ice, and then centrifuged at 12000rpm for 15 min at 4°C. Cell nucleoprotein was extracted using NE-PER™ Nuclear and Cytoplasmic Extraction Reagents (Thermo Fisher Scientific). Protein concentration was determined by BCA assay (Thermo Fisher Scientific), and 20 μg of total protein was separated by 12% SDS-PAGE and subsequently transferred to PVDF membrane. After blocking by 3% BSA, membranes were probed with the following antibodies: anti-CARD11(Proteintech, 21741-1-AP), anti-IKKγ (Cell Signaling Technology, #2685), anti-phosphorylation IKKγ (Cell Signaling Technology, #2689), anti-NF-κB p65(Cell Signaling Technology, #8242), anti-phosphorylation mTOR(Ser2448) (Cell Signaling Technology, #2971), anti-phosphorylation JNK(Tyr185) (Proteintech, 80024-1-RR). Bound antibodies were detected using appropriate HRP-conjugated secondary antibodies (Southern Biotech) and enhanced chemiluminescence (ECL, Thermo Fisher Scientific).

### Immunofluorescence

Cells adhered to poly-L-lysine–coated slides were fixed for 15 min with 3% paraformaldehyde, and then permeabilized for 15 min in 0.1% Triton X-100/PBS. After blocking for 60 min in 3% BSA/PBS, cells spots were incubated with the anti-CARD11 (Proteintech, 21741-1-AP) antibody for 60min. Cells were washed in PBS for 3 times and then incubated with goat anti–rabbit secondary Ab conjugated to Alexa Fluor 488 (Invitrogen) After washing in PBS, coverlips were attached with Fluoromount (SouthernBiotech). Fluorescent images were acquired on a fluorescence microscope using a 100× oil immersion objective.

### IgH Rearrangement Analysis

DNA was extracted from peripheral blood using QIAamp Blood DNA mini kit (Qiagen, Hilden, Germany). PCR was performed using consensus fluorescent labeled primers to V_H_ framework region (FR I and II) and joining region(J_H_) of the IgH gene. PCR products were separated by capillary electrophoresis on an ABI 3500DX Genetic Analyzer with electropherograms analyzed using GeneMapper software version 5.0 (Applied Biosystems).

### Luciferase Reporter Assays

Hela cells were plated in 12-well tissue culture plates (3 × 10^5^ cells/well) and were maintained for 24 h. The cells were then co-transfected with 500 ng of pcDNA3.1+, CARD11-expression vector (CARD11-WT, -C49Y- and -G126D) as needed, 300 ng of pNFκB-luc (Bayotime, Shanghai, China) containing NF-κB binding motifs (GGGAATTTCC), and 100 ng of pRL-TK control plasmid (containing Renilla reniformis luciferase gene, Promega, Madison, WI). After 36-48h, the cells were harvested in lysis buffer and were analyzed for luciferase activity using the Dual-Luciferase reporter Assay System (Promega, Madison, WI). Three independent experiments were performed to assess luciferase activity.

### Natural Killer Cell Activity and CD107a Degranulation Assay

This assay was performed in the Beijing Friendship Hospital. Briefly, whole blood samples (5 mL) were collected from the patient and three age-matched healthy controls in EDTA-containing vials, and PBMCs were isolated. K562 cells stably expressing enhanced green fluorescent protein (EGFP) were constructed as target cells (EGFP-K562), and the effector cells were from healthy controls. The cells were incubated for 4 hours according to the effector/target cell ratio of 10:1, with the density of effector cells of 5×10^6^/ml. Meanwhile, EGFP-K562 cells only were included as the background control. After staining with Annexin V-PE and 7-ADD, NK cell activity was analyzed by evaluating the apoptosis ratio of targets cells after co-culture of effector with target cells. Detailed protocol was described previously (15).

CD107a degranulation assay was performed with the same target and effector cells. PBMCs were isolated and incubated overnight at 37°C in medium. Step by step procedures were according to the articles (16), and data were acquired on a FACSCalibur (BD Biosciences). The ratio of NK cells expressing CD107a were compared between co-cultured with K562 cells and medium alone. The ΔCD107a was defined as the difference in the percentage of NK cells expressing CD107a incubated in different conditions.

### RNA-Seq Analysis

RNA was isolated from Hela cells transfected with *CARD11*-G126D and *CARD11*-WT, respectively, and 1 mg RNA was used for constructing cDNA libraries. The standard Illumina Pipeline for RNA-Seq was used by using paired-end 108-bp runs with each sample run in one sequencing lane, and yielded 20 million reads per sample, and the raw data was submitted to the database (https://submit.ncbi.nlm.nih.gov/subs/sra/SUB9299352/). Gene expression was calculated as transcripts per million (TPM) mapped reads by using the TopHat alignment program with redundant reads removed, and the expression values of reads were normalized using $\text{Lo}{\text{g}}_{2}^{\text{TPM}}$.

### Quantitative Real-Time PCR

Total RNA was extracted from Hela cells and PBMCs isolated from the patient’s and healthy controls by using Trizol Reagent (Invitrogen, USA), respectively. The first complementary DNA was synthesized from RNA using reverse transcriptase (TAKARA, Dalian). Real-time PCR was performed in 7500 instrument (Applied Biosystems) using SYBR Green PCR Kit (TaKaRa, Dalian) and *ACTIN* was included as an internal control. The primer sequences of the genes are shown in **Table 1**.

**Table 1:** The primer sequence of the gene.

Gene	Forward primer	Reverse primer
CARD11	CCAGGACCAATGGTCAAGAAGC	TGATGTTCGCTTCAGGCTGATG
RELB	GATTGCCATTGTGTTCAAGAC	CTTCTTGTCCACGCCGTAGCTG
BCL2	TTTCTTGAAGGTTTCCTCGTCCC	ACAGGCCACGTAAAGCAACTCTC
NFKB2	CTCTGCCTTCCTTAGAGCCAG	CCGAACCTCAATGTCATCTTTC
TNFAIP3	CTCCTCCAGCCTCAGCACCAGC	CAGCCGGCTT TTCTGCACTT GCTC
CCND3	GCCTGCGGGCCTGTCAGGAGCAG	CTGTAGGAGTGCTGGTCTGGCTG
PRDX1	CCCAAGCTGATAGGAAGATGTCTTC	GCACACAAAGGTGAAGTCAAGAG
ACTIN	CACAGTGCTGTCTGGCGGCACCAC	GATGGAGCCGCCGATCCACACGGA

### Statistics Methods

Statistical analyses were performed using Graphpad Prism 5 (Graphpad software, USA). Every experiment was repeated three times with duplicates, and data are reported as the mean ± SD. A Student’s t-test was used to compare two groups, and a one-way analysis of variance (ANOVA) was used when comparing three or more groups and statistical significance is indicated by *P<0.05; **P<0.01, ***P<0.001.

## Results

### Clinical Investigations

Our patient, an 8-month-old Chinese boy, is the only child from healthy nonconsanguineous parents without related personal or familial medical history (**Table 2**). He was born at 39 weeks of gestation by cesarean delivery without complications. When 6-month old, the patient first presented with a persistent upper respiratory tract infection, fever with splenomegaly, lymphadenopathy, and anemia. The highest temperature was 39.1°C. As shown in **Table 2**, clinical laboratory examination showed lymphocytosis with normal morphological lymphocytes and normal total white blood cells, increased glutamic-pyruvic transaminase and aspartate aminotransferase, positive perforin, granzyme, and soluble CD25, and normal CD3-CD56+NK cells of lymphocytes, increased ferritin, lower fibrinogen, and elevated triglycerides. The diagnosis of hemophagocytic lymphohistiocytosis (HLH) was considered according to the clinical characteristics and laboratory examinations.

**Table 2:** Clinical characteristics of the patient.

Clinical manifestation	Detection result	Reference value
Age/sex	8 month/male	
Age of onset	6 month	
Clinical manifestation	fever with splenomegaly, lymphadenopathy	
Glutamic-pyruvic transaminase (U/L)	96.1	9-60
Aspartate aminotransferase (U/L)	196.6	10-50
Ferritin (ng/ml)	2478	22-322
Lymphocytes (%)	85.9	40-70
CD3+ T lymphocytes (n/ul)	1624	805-4459
CD3+%	32.99	38.56-70.06
CD4+ T lymphocytes (n/ul)	648	345-2350
CD4+%	12.67	14.21-36.99
CD8+ T lymphocytes (n/ul)	932	314-2080
CD8+%	18.22	13.24-38.53
CD 19+ B lymphocytes (n/ul)	2882	240-1317
CD 19+ %	60.78	10.86-28.03
CD 16 + 56+ NK cells (n/ul)	372	210-1514
CD 16 + 56+ %	4.17	7.92-33.99
CD 3 + 56+ %	6.11	5-26
s.IgG (g/L)	18.6	3.48-7.01
s.IgA (g/L)	0.11	0.06-0.84
s.IgM (g/L)	0.52	0.26-0.90
s.CD25 (pg/ml)	8730.98	410-2623
Perforin (%)	96.81	>84
Granzyme (%)	87.53	>78
Triglycerides (mmol/L)	1.61	0.32-1.46
Fibrogen (g/L)	1.55	2-4
IL-6 (pg/ml)	12.6	0-3.4
IL-8 (pg/ml)	494	0-62
IL-10 (pg/ml)	122	0-9.1
Bone marrow cell morphology	increased lymphocytes and decreased granulocytes	
Karyotype	46,XY	

**Figure 1:**
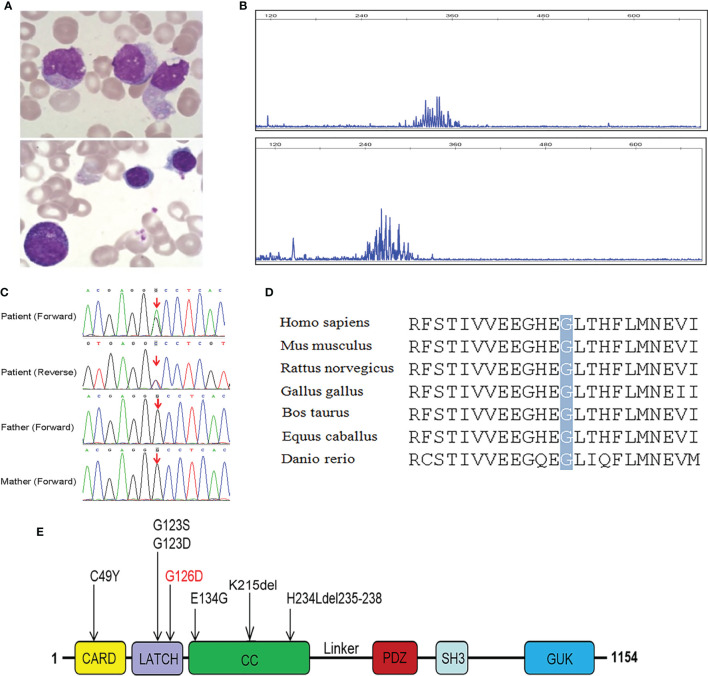
Characterization of our patient with mutated *CARD11* gene and conserved features of CARD11 protein. **(A)** Bone marrow smear from the patient, showed increased lymphocytes and decreased granulocytes; **(B)** Result of IgH rearrangement of our patient, it shows polyclonal rearrangement in FR1-JH region (above) and FR2-JH region (below); **(C)** Sanger sequencing of *CARD11* mutation in the family; **(D)** Conservation analysis of CARD11 protein among different species. The position of the mutations at residue 126 is indicated by a gray bar and highly conserved throughout all indicated species; **(E)** Scheme of the distribution of GOF CARD11 mutations, and the mutation in red was reported in our patient.

In addition, he had a hypergammaglobulinemia, decreased serum IgA levels, and normal IgM levels. Bone marrow smear showed increased lymphocytes (40.5%) and decreased granulocytes (15.5%) (**Figure 1A**). Lymphocyte proliferation in response to phytohemagglutinin (PHA) was poor, and IgH rearrangement indicated that patient B cells are polyclonal (**Figure 1B**).

### WES Analysis and Putative Pathogenic Mutation Screening

In order to identify the pathogenic gene and verify the diagnosis of HLH, trios WES was conducted. Bioinformatic analysis was performed to identify candidate pathogenic gene according to filtering strategy based on variant classification, population frequency, and variant functional damaging prediction, including Sorting Intolerant From Tolerant (SIFT), Polymorphism Phenotyping v2 (PolyPhen-2), and Combined Annotation Dependent Depletion (CADD). Surprisingly, we did not find any pathogenic variant in HLH-associated genes after filtration, but instead one germline heterozygous missense mutation in exon 5 of *CARD11* (NM_032415), c.377G>A (p.G126D) was identified. Sanger sequencing showed that this mutation was *de novo* in the patient and his parents did not carry this mutation (**Figure 1C**). The mutated site is conserved among different species (**Figure 1D**), located within the LATCH domain (Residues 112-130) of CARD11 protein (**Figure 1E**) in human, and has not previously been reported. The novel germline mutation was submitted to the database(https://submit.ncbi.nlm.nih.gov/subs/variation_clinvar/SUB9923934/). The diagnosis of BENTA was made, based on the patient’s clinical presentation: polyclonal B cell lymphocytosis, splenomegaly, lymphadenopathy, and recurrent infection.

### Effects of G126D on CARD11 Cellular Distribution and Activation of NF-κB, JNK, and mTOR Signaling Pathways

**Figure 2:**
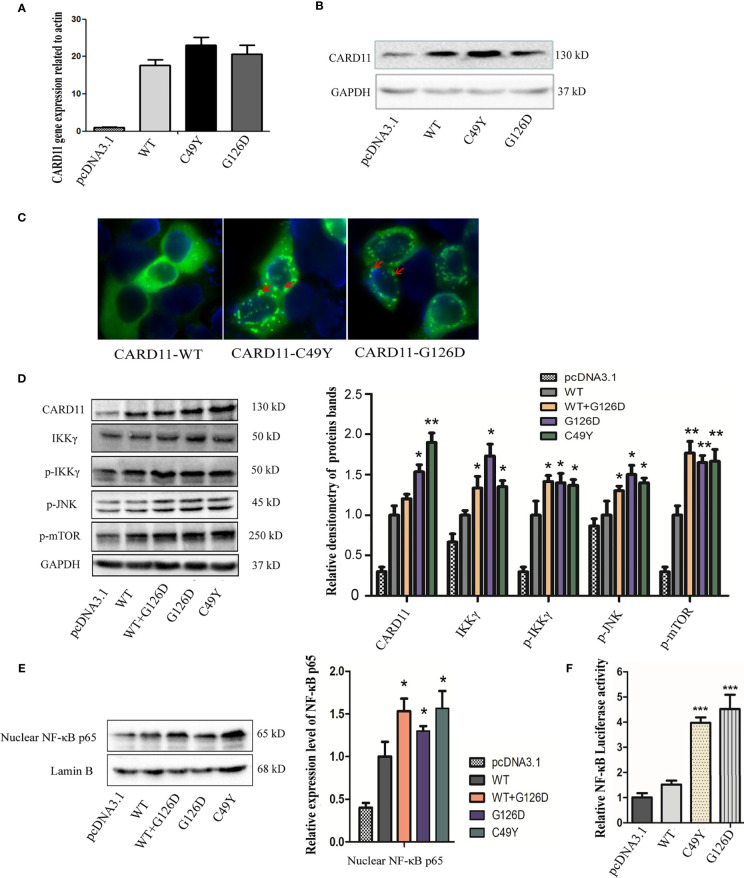
Functional study of G126D mutation in Hela cells. The expression levels of wild-type and mutant CARD11 gene in Hela cells were detected by Real-time PCR **(A)** and western blot **(B)**. **(C)** Distribution of CARD11-WT, -C49Y, and -G126D, respectively, investigated by immunofluorescence. **(D)** The expression levels of CARD11, IKKγ, p-IKKγ anti-NF-κB p65, p-MTOR, p-JNK in Hela cells transfected with wildtype or mutant *CARD11*, respectively, in whole cell lysates. GAPDH serves as a loading control; **(E)** Phosphorylated p65 increased in nuclear lysate of cells with mutant *CARD11*, and lamin B serves as a loading control. **(F)** Activity of NF-κB-dependent luciferase of cell extracts from each sample was measured and recorded as a fold increase compared to control cells with empty pcDNA3.1 plasmid. The results from three independent experiments are expressed as the mean + standard deviation (*P < 0.05; **P < 0.01; ***P < 0.001).

In order to establish the G126D as a pathogenic variant, *in vitro* functional study was performed. WT-CARD11, G126D-CARD11, C49Y-CARD11 (positive control) pcDNA3.1 empty plasmids alone, were transfected into Hela cells, respectively (**Figures 2A, B**). Protein aggregates were observed in cells ectopically expressing C49Y- or G126D-CARD11 proteins, whereas the proteins dispersed throughout cytoplasm in the cells transfected with WT-CARD11 (**Figure 2C**), suggesting that both C49Y and G126D mutations result in aggregation of CARD11 proteins.

As reported previously, CARD11 plays an essential role in NF-κB activation (3). To investigate the effects of G126D mutation, we measured the levels of IKKγ and phosphorylated IKKγ in whole cell protein extracts, as well as NF-κB p65 in the nuclear fraction. We noticed that all of them were significantly increased in mutant CARD11 compared to WT CARD11 (**Figures 2D, E**). In addition, increased soluble CD25 was detected in our patient (**Table 2**).

NF-κB activation was determined by measuring pNF-κB-luc-reporter gene expression in Hela cells transiently transfected with pcDNA3.1, *CARD11*-WT, -G126D, or -C49Y plasmids. As shown in **Figure 2F**, the luciferase activity in cells with mutated CARD11 was significantly higher than that in cells with CARD11-WT.

Taken together, these data indicated that G126D mutation is a GOF mutation and could spontaneously signal and promote NF-κB activation independently of antigen receptor stimulation.

As CARD11 serves as a bridge linking cell surface antigen receptor signaling with the activation of the NF-κB, JNK, and mTORC1 signaling, we also investigated the contribution of CARD11 mutation to the activation of JNK and mTOR pathways by assessing their extent of phosphorylation in Hela cells. As expected, phosphorylation of both JNK and mTOR substantially increased in cells with mutant CARD11compared to that in control cells (**Figure 2D**).

**Figure 3:**
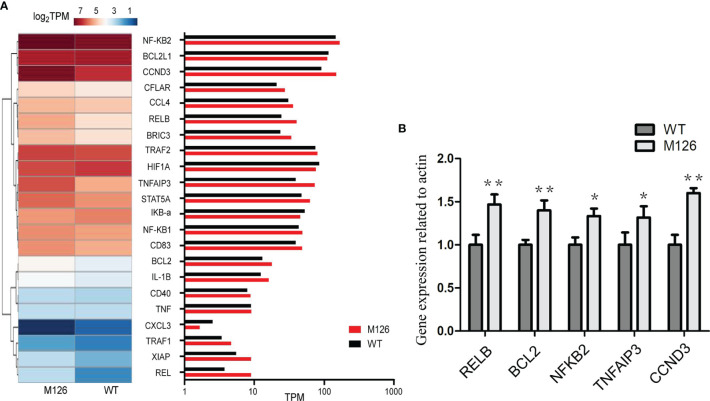
G126D leads to upregulation of some downstream targets of NF-κB signal pathway. **(A)** Heat map of differentially expressed genes involved in NF-κB signal pathway between CARD11-WT and CARD11-G126D by RNA-seq analysis in Hela cells. The color scales of heatmap refer to $\text{Lo}{\text{g}}_{2}^{\text{TPM}}$. **(B)** Realtime PCR was performed to measure the expression level of *RELB, BCL2*, *NFKB2*, *TNFAIP3* and *CCND3* gene in Hela cells. WT, wild type; M126, G126D mutation; TPM, Transcripts per million in RNA seq. The results of three independent experiments are expressed as the mean + standard deviation (*P < 0.05; **P < 0.01).

All these results suggest that G126D is a GOF mutation, and could enhance the expression of downstream effectors such as NF-κB, JNK, and mTORC1. To further examine the effect of G126D on downstream genes, RNA-seq was performed. KEGG pathway analysis showed that some genes involved in NF-κB pathway, were up-regulated in cells with G126D mutation, such as *CCND3*, *RELB* (encoding an NF-κB Subunit), *NFKB2*, and *TNFAIP3* (**Figures 3A, B**), which is consistent with constitutive activation of NF-κB signaling.

### Impact of CARD11 G126D on Natural Killer Cell Activity

**Figure 4:**
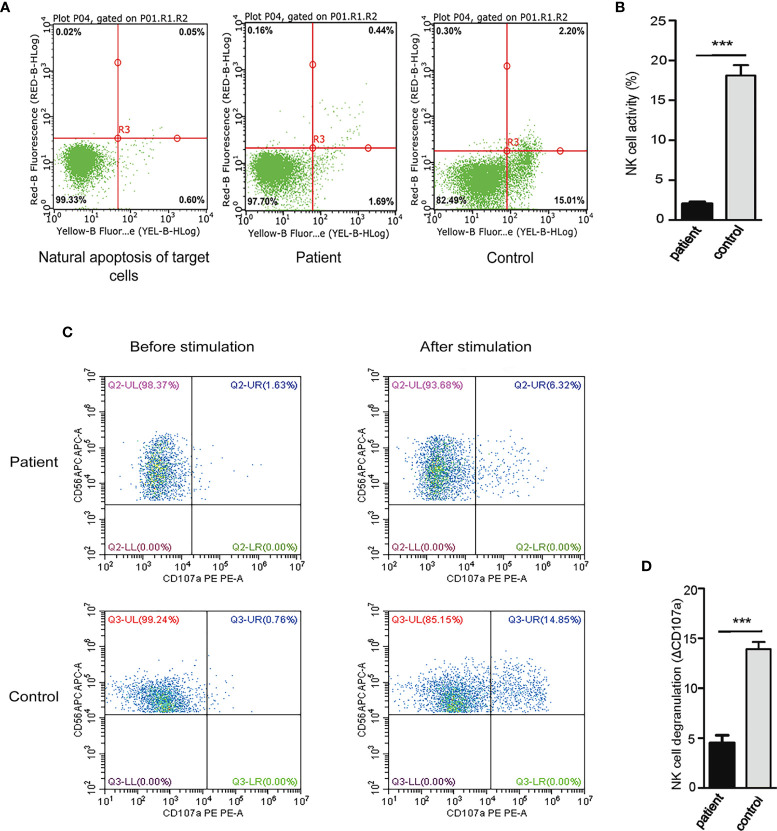
Decreased NK cell activity and degranulation in our patient. **(A)** NK cell activity was measured as the proportion of apoptosis in target cells (EGFP-K562) incubated with NK cells isolated from our patient or normal control, and bivariate distribution was set, with Annexin V-PE and 7-AAD on the horizontal and vertical axes. **(B)** The proportion of apoptosis in EGFP-K562 cells was decreased when incubated with NK cells of this patient compared with that of control. The results showed that the activity of NK cells decreased significantly. **(C)** The NK cells expressing CD107a were compared between these co-cultured with K562 cells and those incubated with medium alone. The ΔCD107a was defined as the difference in the percentage of NK cells expressing CD107a incubated under different conditions. **(D)** ΔCD107a was decreased after stimulation and abnormal degranulation was observed in this patient compared with control, The results suggested degranulation function of NK cells decreased significantly. (***P < 0.001). HD, Healthy donor.

Natural Killer (NK) cells are important effector lymphocytes that are best characterized for their antiviral and anticancer activities. NK cells can directly kill target cells through cytotoxic mechanism. Previous study revealed that constitutive activation of NF-κB driven by mutant CARD11 may be stimulatory in B cells, which contributes to polyclonal B cell lymphocytosis, but partially inhibitory in T cells, which render normal T cells hyporesponsive to antigen receptor stimulation (8). However, it is unclear whether G126D mutation of *CARD11* exerts effects on NK cells, particularly their killing activity. To answer this question, we assayed NK cell activity by measuring the proportion of apoptosis in target cells (EGFP-K562) incubated with NK cells isolated from our patient or WT controls, respectively. Our results showed that NK cell activity in the blood sample from the patient was obviously lower than those from three controls (P<0.001) (**Figures 4A, B**). Furthermore, we evaluated NK activity by measuring the expressions of CD107a, a sensitive marker of NK cell activity, by performing degranulation assays using flow cytometry. Our results showed that the degranulation was dramatically decreased in the patient (P<0.001) (**Figures 4C, D**). Collectively, the G126D mutation of CARD11 is associated with decreased NK cell activity.

**Figure 5:**
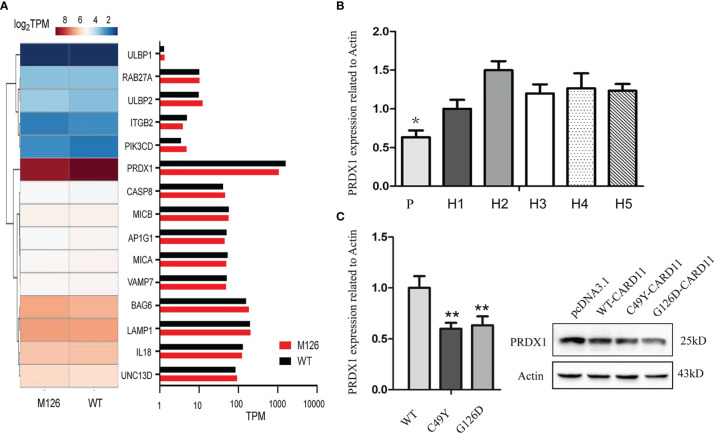
Impact of CARD11 G126D on Natural Killer cell activity. **(A)** Heat map of differentially expressed genes involved in NK cell activation between CARD11-WT and CARD11-G126D, as measured by RNA-seq analysis in Hela cells. The color scales of heatmap refer to $\text{Lo}{\text{g}}_{2}^{\text{TPM}}$. **(B)** Expression level of *PRDX1* gene in peripheral blood by Realtime PCR. **(C)** The mRNA level of *PRDX1* in Hela cells by Realtime PCR and protein level by western blot. P, patient; N, normal controls; WT, wild type; M126, G126D mutation; TPM, Transcripts per million in RNA seq; H, Healthy donor. The results of three independent experiments are expressed as the mean + standard deviation (*P < 0.05; **P < 0.01).

To identify potential mechanisms through which G126D mutation impaired NK cell activity, we analyzed the expression of genes involved in NK cell activity and degranulation by Gene Ontology (GO) enrichment analysis. We found significant differences in several genes, such as *PRDX1* (peroxiredoxins), *BAG6*, *IL18*, and *ITGB2* (**Figure 5A**). The expression of *PRDX1*, a gene positively regulating the activation and functions of NK cells, had the most significant decrease. Further, real-time PCR confirmed the decreased expression of *PRDX1* gene in peripheral blood of this patient (**Figure 5B**) and in mutant Hela cells (**Figure 5C**) compared to their respective controls.

## Discussion

In this study, we reported an 8-month-old patient with B cell lymphocytosis, fever, splenomegaly, lymphadenopathy, and recurrent infection, who was initially diagnosed as HLH. However, WES revealed a *de novo* germline missense mutation, G126D in *CARD11* gene, which was associated SCID, BENTA, and severe atopic disease. Based on his clinical manifestations and the genetic findings, a diagnosis of BENTA was considered and HLH could be secondary to BENTA. This is the first report of BENTA disease in Chinese population.

One previous study reported that a patient with BENTA showed HLH presentations at the terminal stage of illness and received treatment following the HLH-2004 protocol (9), but the patient did not respond to the treatment and died at the age of 3.5 years. The patient in our study only received intravenous antibiotics for recurrent respiratory tract infections and blood transfusion due to severe anemia. His clinical symptoms were mild and did not practice a formal HLH treatment, but dexamethasone was taken orally for maintenance therapy.

Ever since the first case of gain-of-function *CARD11* mutation was described in 2012 (8), a total of 14 BENTA disease patients with six different germline heterozygous mutations (C49Y,G123S, G123D, E134G, K215del, and H234Ldel235-8) have been reported (8–13) and all of the *CARD11* GOF mutations are heterozygous missense mutations within the N-terminal CARD, LATCH, and CC domains. No GOF mutation was detected in the C-terminal domains.

CARD11 protein is required for BCR- and TCR-mediated activation of IKK complex, which in turn phosphorylates IkB (inhibitor of NF-κB), and leads to the activation of NF-κB (14, 17). Following BCR or TCR engagement, CARD11 undergoes a conformational change from an inactive state to an active scaffold, and the latent state is controlled by ID domain, the domain between CC and PDZ domains (18, 19). In addition, LATCH domain not only interacts with the CARD to promote CARD11 autoinhibition, but also plays a critical role in controlling the interaction of CARD11 with the adapter, Bcl10 (7, 20). Therefore, mutations in these domains may bypass common controls and induce NF-κB activation by disrupting CARD11 autoinhibition.

The CARD11 G126D mutation promotes the activation of JNK and mTOR as CARD11 is also a multidomain signaling scaffold protein required for antigen receptor signaling to c-Jun and mTOR (19).

Somatic G126D mutation was reported in two patients with diffuse large B-cell lymphoma (DLBCL) (21, 22). Moreover somatic G126D mutation also was a GOF mutation by using the library of murine CARD11 variants containing random mutations (21). However, to our knowledge, this variant has not been reported in germline cells. In order to further clarify the function of G126D and to make a definitive diagnosis, we performed *in vitro* experiments to evaluate its possible effects and underlying mechanisms. Our findings suggested that G126D changes the distribution pattern of CARD11 from dispersion to aggregation and increases activation of NF-κB, which is consistent with previous studies on GOF mutations within CARD, LATCH, and CC domains at the N terminus (13).

Significant B cell lymphocytosis and impaired T cell proliferation were reported in BENTA patients, which are associated with B cell malignancy and recurrent infection (19). Previous study has showed that a DN CARD11 mutation (R30W) impaired inflammatory cytokine (e.g. IFN-γ) production by NK cells, but not affecting NK cell cytotoxicity (23). However, the impacts of GOF CARD11 mutations on NK cell functions are unknown.

Therefore, we attempted to investigate NK cell activity by examining the apoptosis of EGFP-K562 target cells incubated with NK cells from the patient, and the surface expression of CD107a (24). Our study showed decreased NK cell activity in our patient compared to control. However, the absolute number of NK cells in our patient was not increased compensatory but remained at normal range (in our patient) or slightly lower level (9, 17). Therefore, the whole NK cell activity in BENTA patient was declined. It is well known that NK cells have a critical role in innate and adaptive immune against malignant transformation and viral infection (25, 26), so impaired NK cell activity might enhance the risk of cancer and infection.

Amongst the differently expressed genes, *PRDX1* was the most significantly decreased. PRDX1 enhances the cytotoxicity of NK cells by balancing redox in the NK cells (27, 28). It was reported that dysfunction of PRDX-related antioxidant chain led to profound alterations in spontaneous and antibody-dependent NK cell cytotoxicity, impaired degranulation, and decreased activation (29). Prdx1-null mice have abnormalities in the total number, relevant phenotypes, and function of natural killer cells (30). Therefore, PRDX11 could play a critical role in total NK cell activity in BENTA patient with CARD11 G126D mutation, and the detailed mechanism warrants further study.

In conclusion, we identified and characterized a *de novo* germline heterozygous GOF variant in *CARD11* gene from a patient with B cell lymphocytosis and constitutive NF-κB activation. Our study emphasizes the importance of WES in assisting with the diagnosis of rare immune diseases and repeated infectious diseases, and also provides functional evidence of pathogenicity of G126D mutation in *CARD11* gene.

## Data Availability Statement

The datasets presented in this study can be found in online repositories. The names of the repository/repositories and accession number(s) can be found below: SRA PRJNA743711, ClinVar SCV001739273.

## Ethics Statement

The studies involving human participants were reviewed and approved by The institutional review board of Wuhan Children’s Hospital, Tongji Medical College, Huazhong University of Science & Technology. Written informed consent to participate in this study was provided by the participants’ legal guardian/next of kin. Written informed consent was obtained from the minor(s)’ legal guardian/next of kin for the publication of any potentially identifiable images or data included in this article.

## Author Contributions

Study concepts: XH, HX, and AZ. Study design: PZ, QM, AZ, and HX. Literature research: YH, QM, and PZ. Clinical information collection: YH, HX, and QM. Data acquisition: QM, YH, LZ, and SL. Data analysis/interpretation: YH, QM, XZ, and LT. Manuscript preparation: XH and PZ. Manuscript editing: XH. Manuscript revision/review: AZ and HX. Manuscript final version approval: HX and AZ. All authors contributed to the article and approved the submitted version.

## Funding

This work was supported by the grants of Wuhan Municipal Health Commission (NO. WX19C19, WX14A06); Youth Program of National Natural Science Foundation of China (NO.81700302); Natural Science Foundation of Hubei Province (2017CFB322).

## Conflict of Interest

The authors declare that the research was conducted in the absence of any commercial or financial relationships that could be construed as a potential conflict of interest.

## Publisher’s Note

All claims expressed in this article are solely those of the authors and do not necessarily represent those of their affiliated organizations, or those of the publisher, the editors and the reviewers. Any product that may be evaluated in this article, or claim that may be made by its manufacturer, is not guaranteed or endorsed by the publisher.
